# Emergence of vaccine-derived poliovirus type 2 after using monovalent type 2 oral poliovirus vaccine in an outbreak response, Philippines

**DOI:** 10.5365/wpsar.2022.13.2.904

**Published:** 2022-05-25

**Authors:** SweetC B Alipon, Yoshihiro Takashima, Tigran Avagyan, Varja Grabovac, Syeda Kanwal Aslam, Benjamin Bayutas, Josephine Logronio, Xiaojun Wang, Achyut Shrestha, Sukadeo Neupane, Maria Concepcion Roces, Lea Necitas Apostol, Nemia Sucaldito

**Affiliations:** a Vaccine-Preventable Diseases and Immunization, Division of Programmes for Disease Control, World Health Organization Regional Office for the Western Pacific, Manila, Philippines.; b World Health Organization Representative Office for the Philippines, Manila, Philippines.; c Research Institute for Tropical Medicine, Department of Health, Manila, Philippines.; d Public Health Surveillance Division, Department of Health, Manila, Philippines.

## Abstract

**Objective:**

In response to an outbreak of circulating vaccine-derived poliovirus (cVDPV) type 2 in the Philippines in 2019–2020, several rounds of supplementary immunization activities using the monovalent type 2 oral poliovirus vaccine (OPV) were conducted for the first time in the Western Pacific Region. After use of the monovalent vaccine, the emergence of vaccine-derived poliovirus unrelated to the outbreak virus was detected in healthy children and environmental samples. This report describes the detection of this poliovirus in the Philippines after use of the monovalent type 2 OPV for outbreak response.

**Methods:**

We describe the emergence of vaccine-derived poliovirus unrelated to the outbreak detected after supplementary immunization activities using the monovalent type 2 OPV. This analysis included virus characterization, phylogenetic analyses and epidemiological investigations.

**Results:**

Three environmental samples and samples from six healthy children tested positive for the emergent vaccine-derived poliovirus. All isolates differed from the Sabin type 2 reference strain by 6–13 nucleotide changes, and all were detected in the National Capital Region and Region 4, which had conducted supplementary immunization activities.

**Discussion:**

Since the 2016 removal of type 2 strains from the OPV, vaccine-derived poliovirus outbreaks have occurred in communities that are immunologically naive to poliovirus type 2 and in areas with recent use of monovalent OPV. To prevent the emergence and further spread of cVDPV type 2, several interventions could be implemented including optimizing outbreak responses by using the monovalent type 2 OPV, accelerating the availability of the novel type 2 OPV, strengthening routine immunization using inactivated polio vaccine and eventually replacing OPV with inactivated poliovirus vaccine for routine immunization.

Poliomyelitis is an acute viral infection of the nervous system caused by poliovirus types 1, 2 and 3. Polio has been eliminated in most countries globally through vaccination. Wild poliovirus type 2 was last seen in 1999 and was certified as eradicated in 2015. Oral poliovirus vaccine (OPV) remains the vaccine of choice for global polio eradication due to its ability to interrupt transmission of poliovirus by inducing mucosal immunity. (*1*) However, in underimmunized populations, the weakened vaccine virus from OPV may genetically mutate from the original attenuated strain and regain its neurovirulence, causing paralysis and outbreaks. Among the three Sabin strains in the OPV, before 2016 type 2 was estimated to cause 40% of all vaccine-associated paralytic polio and 90% of all cases of circulating vaccine-derived poliovirus (cVDPV). (*2*)

In April 2016, the poliovirus type 2 Sabin strain was removed from the trivalent OPV during the global switch to bivalent OPV to stop the emergence of VDPV from poliovirus type 2. (*3*) The inactivated poliovirus vaccine (IPV) had been introduced, but it provided only limited mucosal immunity to stop the spread of poliovirus, and at the time of the switch, there was a severe shortage of the IPV so that large cohorts of newborns were left unvaccinated. (*4*) As a result, the number of outbreaks from cVDPV type 2 (cVDPV2) has been increasing due to large gaps in population immunity to poliovirus type 2. (*5*) The expanding global cohort of children without the immunity against poliovirus type 2 that would prevent transmission could result in established endemicity of VDPV. (*6*)

To combat the growing threat of cVDPV2, several interventions could be implemented including optimizing outbreak responses by using the monovalent type 2 OPV, strengthening routine immunization by using IPV and accelerating the availability of the novel type 2 OPV. However, use of the monovalent type 2 OPV to control outbreaks of cVDPV2 carries the risk of seeding emergent strains of VDPV2 that have the potential for further circulation. (*7*) This has been observed through molecular epidemiological analysis of cVDPV2 outbreaks that resulted from suboptimal coverage of supplementary immunization activities (SIAs) that used the monovalent type 2 OPV within outbreak response zones or in contacts travelling outside of response zones. (*6*, *7*) Therefore, use of the monovalent type 2 OPV in outbreak responses is governed by the strict criteria of the protocol of the Global Polio Eradication Initiative (GPEI) and the decision to release the monovalent type 2 OPV from global stocks, authorized by the Director-General of the World Health Organization (WHO) based on the recommendations of the GPEI’s Eradication and Outbreak Management’s advisory group.

On 19 September 2019, a polio outbreak was declared by the Department of Health in the Philippines after confirmation of cVDPV2 in a child with acute flaccid paralysis (AFP) that was reported from Lanao Del Sur Province in the Bangsamoro Autonomous Region of Muslim Mindanao (BARMM). The index child was a 3-year-old girl with no history of polio vaccination and onset of paralysis on 26 June 2019. Poliovirus collected from stool in July 2019 had 65 nucleotide changes from the Sabin type 2 reference strain and was genetically linked to isolates collected from July to August in environmental samples in Manila, National Capital Region (NCR) and Davao (Mindanao), confirming widespread circulation of VDPV2 within the Philippines.

From June 2019 to March 2021, a further 20 stool samples from 13 AFP cases, two contacts of AFP cases and five healthy children, plus 23 environmental samples tested positive for cVDPV2. Geographically, this outbreak occurred in the Luzon and Mindanao groups of islands, with concentrated virus detection in BARMM and NCR and other regions, including Regions 3, 7, 10, 11 and 12. Analyses revealed that these isolates were genetically linked to one another, and had between 61 and 71 nucleotide changes from the Sabin type 2 reference strain. The last cVDPV2 isolate from a human came from a stool sample from a 1-year-old child from Cabanatuan City, Nueva Ecija, who had onset of paralysis on 15 January 2020. The last cVDPV2 isolate detected in an environmental sample was collected on 16 January 2020 from the Butuanon River in Mandaue City, Region 7.

Along with the cVDPV2 outbreak, VDPV2 was isolated in August 2019 from stool samples from an AFP case with a primary immunodeficiency disorder residing in Laguna, Region 4A. Genetic analysis showed 64–107 nucleotide changes for this isolate compared with the Sabin type 2 reference strain, but it was not genetically linked with any other isolates from other sources in the country.

The use of the monovalent type 2 OPV for the cVDPV2 outbreak response in the Philippines was approved on 24 September 2019. From October 2019 to December 2020, 15 SIAs were completed in outbreak-affected areas, using more than 13 million doses of monovalent type 2 OPV and achieving coverage of 79% to 102% (**Fig. 1**). However, within 30–120 days of monovalent type 2 OPV use, isolates of emergent VDPV2 were detected in several areas where the outbreak response had taken place, and these had between 6 and 13 nucleotide changes from the Sabin type 2 reference strain, which suggests the emergence of a new strain. As there was no evidence of circulation, they were classified as ambiguous VDPV2.

**Figure 1:**
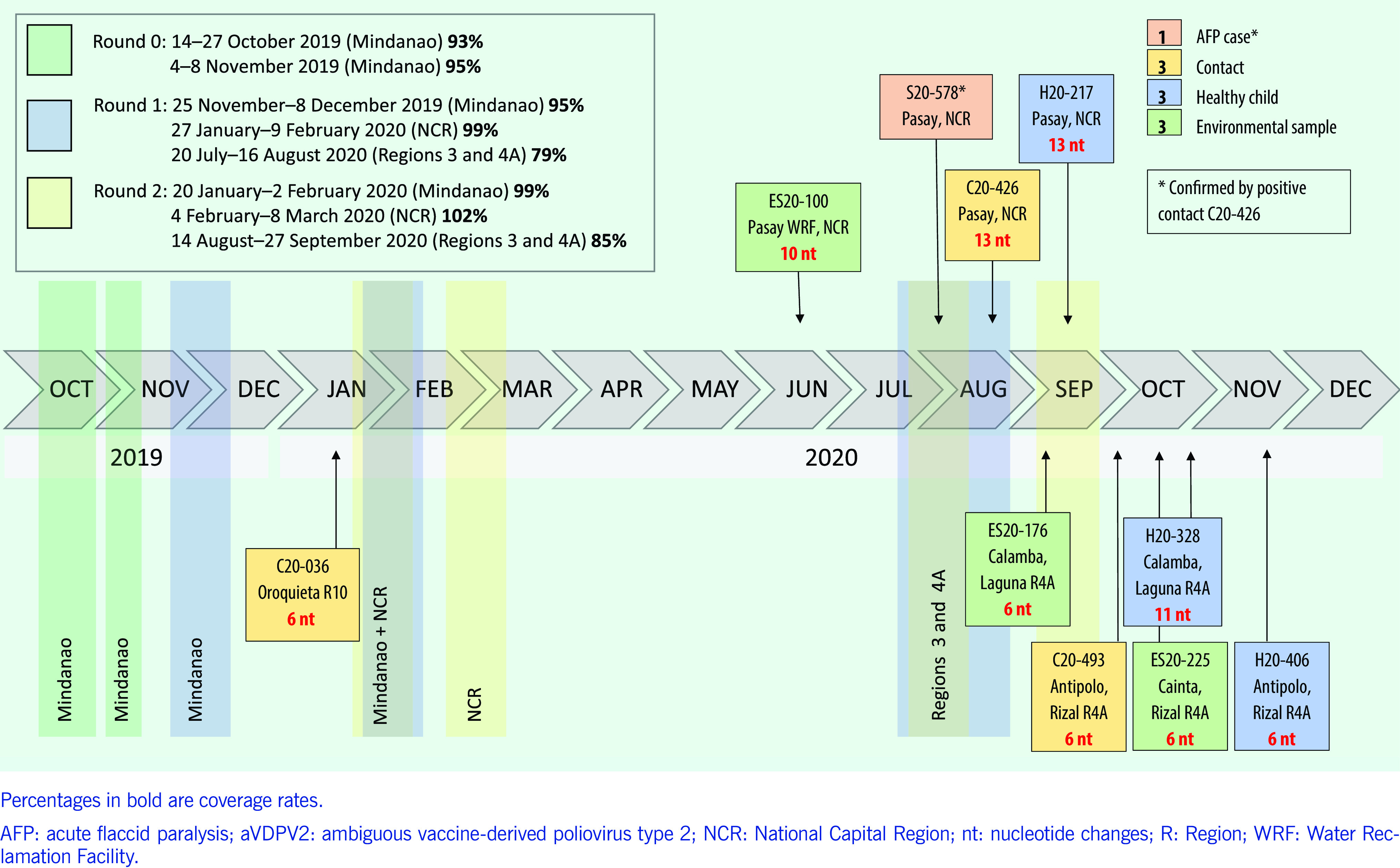
Timeline of supplementary immunization activities using the monovalent type 2 oral poliovirus vaccine and identification of ambiguous isolates of vaccine-derived poliovirus type 2, the Philippines, 2019–2020

[**insert** Figure 1]

This report summarizes the emergence of VDPV2 following use of the monovalent type 2 OPV to respond to the cVDPV2 outbreak in the Philippines, and it contributes to the regional and global knowledge about and experience of the risks related to the use of monovalent type 2 OPV and possible preventive and mitigation activities.

## Methods

The polio surveillance system in the Philippines follows the GPEI protocol and includes AFP surveillance conducted by the Epidemiology and Surveillance Units of the Department of Health. (*8*) This is supplemented by environmental surveillance conducted by the polio team at the Research Institute for Tropical Medicine, whereby environmental samples are collected from all 17 regions. (*9*) At the Research Institute, all samples undergo testing for concentration of sewage, virus culture and intratypic differentiation for serotyping of polioviruses. Every poliovirus type 2 isolate and discordant (non-Sabin) poliovirus types 1 and 3 isolates are sent to the National Institute of Infectious Diseases in Japan for phylogenetic analyses using standardized WHO methods. (*10*) Samples are sequenced and phylogenetic trees are developed to determine the genetic linkage of the polioviruses and their relatedness to the Sabin strain and to one another. Using a global database of known cVDPVs, the genetic linkage of newly detected VDPVs to known VDPVs can be determined. We analysed demographic, clinical and laboratory information recorded in the polio surveillance database and describe the emergent VDPV2 isolates in relation to the timeline of the SIAs that used the monovalent type 2 OPV (**Fig. 1**). This analysis included findings from virus characterization, phylogenetic analyses and epidemiological investigations.

## Results

**Figure 2:**
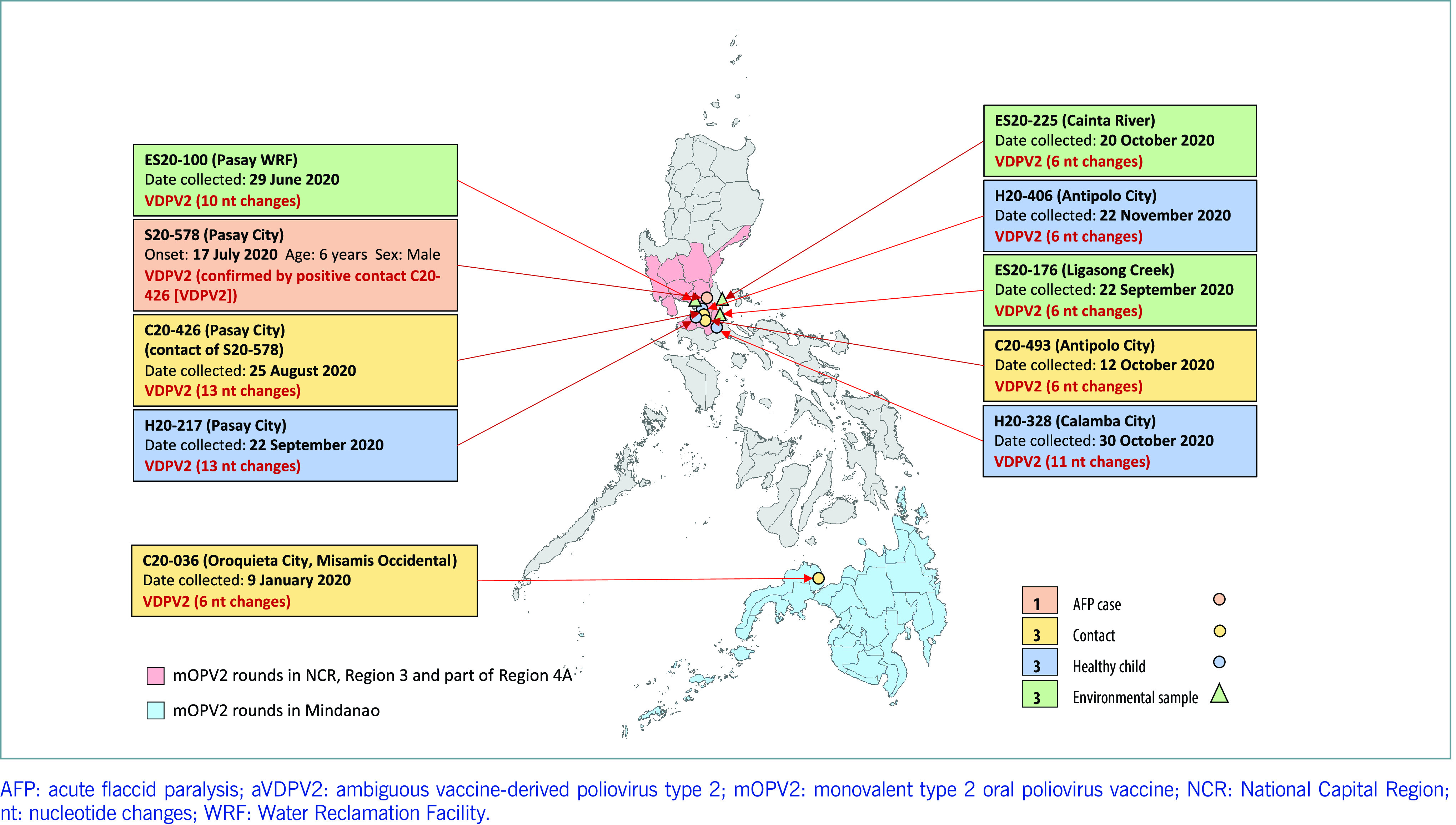
ESpot map of ambiguous vaccine-derived poliovirus type 2, the Philippines, 2019–2020

From October 2019 to December 2020, three environmental samples and six healthy children tested positive for new VDPV2 unrelated to the outbreak virus. All isolates had between 6 and 13 nucleotide changes from the Sabin type 2 reference strain, and all were detected in NCR and Region 4, the regions that conducted SIAs using monovalent type 2 OPV (**Fig. 2**).

[**insert** Figure 2]

The first emergent VDPV2 strain was detected in stool from a close contact of an AFP case in Misamis Occidental Province, Region 10, in January 2020. This was within 60 days of the first SIA delivering monovalent type 2 OPV in Mindanao in 2019. The case was a 3-year-old girl who received her first dose of the monovalent type 2 OPV during the SIA in November 2019. Genetic analysis of the isolate showed six nucleotide changes from the Sabin type 2 reference strain and revealed no genetic link to other cVDPV2 isolates.

The second emergent VDPV2 isolate was detected in an environmental sample from Pasay City, NCR, in June 2020. This was within 120 days of the SIAs using the monovalent type 2 OPV in NCR. The virus had 10 nucleotide changes from the Sabin type 2 reference strain and was not genetically linked to other known VDPV2 isolates. Following this detection, further investigations were conducted, including searching for AFP cases, conducting a household survey and collecting stool samples from healthy children in high-risk barangays (the smallest administrative unit in the Philippines) in the catchment area of the environmental surveillance site in Pasay City. These case-finding efforts identified a 6-year-old boy with paralysis whose stool samples were negative for poliovirus due to inadequate samples; however, the stool sample collected from his close contact, a 1-year-old boy, tested positive for VDPV2 and had 13 nucleotide changes from the Sabin type 2 reference strain. The close contact had received two doses of the monovalent type 2 OPV during the SIAs in January and February 2020. In the high-risk barangays neighbouring the barangay with the AFP case, stool samples were collected from healthy children, and a 7-month-old boy was identified who also tested positive for VDPV2 and whose sample had 13 nucleotide changes from the Sabin type 2 reference strain. This child had received one dose of the monovalent type 2 OPV during the SIA in March.

Similarly, emergent VDPV2 isolates with six nucleotide changes from the Sabin type 2 reference strain were detected in environmental samples in Calamba, Laguna, in Region 4 in September 2020, within 30 days of the SIA using the monovalent type 2 OPV in Laguna Province. Heightened AFP surveillance was conducted in the city, which led to the detection of emergent VDPV2 in the stool sample of a healthy 2-year-old child. This child had received two doses of the monovalent type 2 OPV during the SIAs in Laguna in August and September 2020, and a stool sample was positive for VDPV2, and had 11 nucleotide changes from the Sabin type 2 reference strain, within 45 days after the last dose of the monovalent type 2 OPV.

In Antipolo, Rizal, a close contact of an AFP case tested positive for VDPV2 in November 2020. The close contact was a 2-year-old boy who had received two doses of the monovalent type 2 OPV during the SIAs in August and September 2020 in Rizal Province, Region 4. The isolated poliovirus had six nucleotide changes from the Sabin type 2 reference strain. This case triggered a household survey of healthy children in the affected barangay and other high-risk barangays. A healthy 1-year-old child who had not received any doses of the monovalent type 2 OPV during an SIA tested positive for VDPV2, and the isolate had six nucleotide changes.

Lastly, in a neighbouring municipality in Cainta, Rizal Province, emergent VDPV2 was detected in an environmental sample from the Cainta River in October 2020, within 60 days after the SIA using the monovalent type 2 OPV; the environmental isolate had six nucleotide changes. A summary of the isolates is given in **Tables 1** and **2**.

**Table 1:** Isolates of emergent vaccine-derived poliovirus type 2 collected from children in the Philippines, 2019–2020

Case no. (case identifier)	Age, sex	City, region	No. of OPV or IPV doses received	Date of stool collection	Result	No. of nucleotide changes from the Sabin type 2 reference strain
1 (C20–036)	3 years, female	Oroquieta City, Misamis Occidental	5	9 January 2020	aVDPV2	6
2 (S20–578)	6 years, male	Pasay City, National Capital Region	3	1: 12 August 2020 2: 13 August 2020	Both negative	Close contact (C20–426) was positive
3 (C20–426)	1 year, male	Pasay City, National Capital Region	5	25 August 2020	aVDPV2	13
4 (H20–217)	7 months, male	Pasay City, National Capital Region	1	22 September 2020	aVDPV2	13
5 (C20–493)	2 years, male	Antipolo City, Rizal	4	12 October 2020	aVDPV2	6
6 (H20–328)	2 years, male	Calamba City, Laguna	3	30 October 2020	aVDPV2	11
7 (H20–406)	1 year, male	Antipolo City, Rizal	2	22 November 2020	aVDPV2	6, 7

aVDPV2: ambiguous vaccine-derived poliovirus type 2; IPV: inactivated poliovirus vaccine; OPV: oral poliovirus vaccine.

**Table 2.** Isolates of emergent vaccine-derived poliovirus type 2 collected in environmental samples, the Philippines, 2020

**Table:** 

Sample no. (sample identifier)	Site	Round no. and date of supplementary immunization activity using monovalent type 2 oral poliovirus vaccine in area	Date of collection	Result	No. of nucleotide changes from the Sabin type 2 reference strain
1 (ES20–100)	Pasay Water Reclamation Facility Pasay City, National Capital Region	1: 27 January–8 February 2020 2: 24 February–7 March 2020	29 June 2020	aVDPV2	10
2 (ES20–176)	Ligasong Creek Calamba City, Laguna	1: 3–30 August 2020 2: 14–27 September 2020	22 September 2020	aVDPV2	6
3 (ES20–225)	Cainta River Cainta, Rizal	1: 24 August–6 September 2020 2: 14 September–1 October 2020	20 October 2020	aVDPV2	6

aVDPV2: ambiguous vaccine-derived poliovirus type 2.

## Discussion

In response to the cVDPV2 outbreak in the Philippines that comprised 20 cVDPV2 cases and contacts, 15 SIAs were conducted between October 2019 and December 2020 using the monovalent type 2 OPV. More than 13 million doses of the monovalent type 2 OPV were used. However, within 60–120 days of some of these SIAs, the emergent VDPV2 isolates were detected in the areas targeted by the outbreak response. These isolates were identified in the close contact of a child with paralysis, six healthy children and three environmental samples. All isolates had between 6 and 13 nucleotide changes from the Sabin type 2 reference strain and no genetic linkage to previously detected VDPVs in the Philippines.

Outbreaks of cVDPV are caused when the live, attenuated virus used in vaccines regains its neurovirulence, particularly in settings with chronically low coverage of routine and supplementary polio immunization or in immunodeficient individuals. (*11*) The risk of further cVDPV will persist while any of the three Sabin strains are used for vaccination, either in the bivalent OPV or the monovalent type 2 OPV. Of the three types of VDPVs, the risk of cVDPV2 outbreaks is highest because more than 3 years have passed since cessation of the use of the Sabin 2 vaccine strain, which has led to a decrease in mucosal immunity against type 2 poliovirus. Any VDPV2 emergence has the potential to cause outbreaks in populations that are immunologically naive to poliovirus type 2. A similar situation was observed in Central and Western Africa in 2019, where VDPV2 cases primarily affected type 2-naive children born after the switch from trivalent OPV to bivalent OPV. (*7*)

This cycle of polio associated with VDPV is likely to continue when a cVDPV2 outbreak response uses the monovalent type 2 OPV to interrupt transmission. The detection of emergent VDPV2 in the Philippines should serve as a warning not only for the Philippines but also for other countries with suboptimal coverage of routine polio immunization.

The risk of future cVDPV2 outbreaks appears to be a closer reality, given the scenario of fading type 2 immunity in OPV-using countries coupled with recent use of the monovalent type 2 OPV. In fact, the risk of a cVDPV outbreak is inevitable while there remain subpopulations with chronically low coverage of polio immunization and the use of any type of OPV continues in routine and supplementary polio immunization activities.

Live OPVs remain the workhorses of polio eradication programmes due to their ability to interrupt transmission. Since the removal of type 2 poliovirus from the OPV in 2016, the majority of cVDPV2 outbreaks reported globally have been detected in areas that recently used the monovalent type 2 OPV or in areas that border those where the monovalent type 2 OPV was used, reflecting the risk of VDPV2 emergence after the Sabin type 2 vaccine strain was used in the period after the vaccine changed. (*6*) To prevent the emergence and further spread of cVDPV2, several interventions could be implemented, including optimizing responses to outbreaks by using the monovalent type 2 OPV, strengthening routine immunization using IPV, accelerating the availability of the novel type 2 OPV and eventually replacing OPV with IPV for routine immunization after carefully considering epidemiological and programmatic aspects. This report summarizes the findings of the investigation into the emergence of a VDPV2 outbreak in the Philippines that occurred after the monovalent type 2 OPV was used during 2019–2020, and it adds to the growing global evidence of VDPV2 emergence in the period after the vaccine changed.
